# Using theories and frameworks to understand how to reduce low-value healthcare: a scoping review

**DOI:** 10.1186/s13012-021-01177-1

**Published:** 2022-01-20

**Authors:** Gillian Parker, Nida Shahid, Tim Rappon, Monika Kastner, Karen Born, Whitney Berta

**Affiliations:** 1 Institute of Health Policy, Management and Evaluation, University of Toronto, 155 College Street, 4th Floor, Toronto, Ontario M5T 3M6 Canada; 2 Centre for Research and Innovation, North York General Hospital, 4001, Leslie Street, Toronto, Ontario M2K 1E1 Canada

**Keywords:** Low-value care, Theory, Framework, De-implement, De-adopt, Scoping review

## Abstract

**Background:**

There is recognition that the overuse of procedures, testing, and medications constitutes low-value care which strains the healthcare system and, in some circumstances, can cause unnecessary stress and harm for patients. Initiatives across dozens of countries have raised awareness about the harms of low-value care but have had mixed success and the levels of reductions realized have been modest. Similar to the complex drivers of implementation processes, there is a limited understanding of the individual and social behavioral aspects of de-implementation. While researchers have begun to use theory to elucidate the dynamics of de-implementation, the research remains largely atheoretical. The use of theory supports the understanding of how and why interventions succeed or fail and what key factors predict success. The purpose of this scoping review was to identify and characterize the use of theoretical approaches used to understand and/or explain what influences efforts to reduce low-value care.

**Methods:**

We conducted a review of MEDLINE, EMBASE, CINAHL, and Scopus databases from inception to June 2021. Building on previous research, 43 key terms were used to search the literature. The database searches identified 1998 unique articles for which titles and abstracts were screened for inclusion; 232 items were selected for full-text review.

**Results:**

Forty-eight studies met the inclusion criteria. Over half of the included articles were published in the last 2 years. The Theoretical Domains Framework (TDF) was the most commonly used determinant framework (*n* = 22). Of studies that used classic theories, the majority used the Theory of Planned Behavior (*n* = 6). For implementation theories, Normalization Process Theory and COM-B were used (*n* = 7). Theories or frameworks were used primarily to identify determinants (*n* = 37) and inform data analysis (*n* = 31). Eleven types of low-value care were examined in the included studies, with prescribing practices (e.g., overuse, polypharmacy, and appropriate prescribing) targeted most frequently.

**Conclusions:**

This scoping review provides a rigorous, comprehensive, and extensive synthesis of theoretical approaches used to understand and/or explain what factors influence efforts to reduce low-value care. The results of this review can provide direction and insight for future primary research to support de-implementation and the reduction of low-value care.

Contributions to the literature

- The Theoretical Domains Framework (TDF) is the most commonly used framework for de-implementation.
- Theories and frameworks are primarily used to identify barriers and facilitators and analyze data.
- The majority of included studies were published within the last 2 years.
- Over 75% of the included studies used theory and frameworks to study efforts to reduce inappropriate prescribing, imaging, and laboratory testing.

## Background

The reduction of low-value care is necessary to ensure patient safety, reduce costs, and promote a sustainable healthcare system [1, 2]. Patients can be harmed by low-value care, whether directly, by downstream effects, or by over-testing or over treatment [2, 3]. In addition to harm, it is estimated that 30% of current healthcare dollars, both in Canada and in the USA, are spent on harmful or wasteful practices [4, 5]. Low-value care can be tests, treatments, medications, or procedures in any healthcare setting (e.g., hospital, primary care, long-term care, or public health) which have been deemed, through evidence, to be ineffective, harmful, or unnecessary. Awareness has increased regarding the prevalence of low-value care in the healthcare system and the high frequency with which these practices are used.

In recent years, a number of initiatives have been created, such as the Choosing Wisely campaigns, which seek to identify, raise awareness of, and address the prevalence of low-value care. International Choosing Wisely campaigns, currently in 25 countries worldwide including Brazil, Wales, and Korea, have gained considerable traction with healthcare organizations and providers and have also been the subject of an increasing amount of health services research [6, 7]. The campaigns have identified prevalent practices, such as imaging for low back pain, benzodiazepines for insomnia in older adults, and pre-operative testing for low-risk surgical procedures, which provide evidence and guidance for healthcare providers and organizations endeavoring to reduce low-value care [8]. Researchers have noted that, as a result of the Choosing Wisely campaigns, efforts to identify and prioritize low-value care have increased exponentially but these efforts far exceed research to evaluate the efficacy of clinical and policy initiatives and interventions to reduce these practices [9, 10].

De-implementation—reducing or stopping the use of a health service or practice provided to patients by healthcare practitioners and healthcare delivery systems [11]—is an emerging area of study in healthcare. Numerous researchers have called for increased attention to understanding the process of de-implementation and determining the most effective and efficient strategies to create and sustain this type of practice change [9–16]. Efforts to reduce low-value care can be hindered by practice patterns and habits that can drive the overuse of procedures, medications, and medical devices; overuse is often a consequence of routines or protocols that are embedded in systems which can be difficult to change or alter [17–20]. The acknowledgement of these complexities has caused many to argue for the use of theory to understand de-implementation and to develop effective strategies to de-implement low-value care [10, 12, 18, 21, 22].

While de-implementation research, intervention design, and evaluation has just begun to use theory, more mature areas, such as implementation research, have recognized the value in using theory to design behavior change interventions [15, 23–25]. Implementation and de-implementation research both endeavor to identify what interventions work, for whom and under what circumstances [10]. Grimshaw et al. state that implementation science theories and frameworks can support de-implementation as, they argue, the effectiveness of these theories is a function of an effective process rather than unidentified factors that may reduce intervention effectiveness [10]. Implementation science knowledge, theories, and frameworks can provide support and guidance for de-implementation efforts.

Research with underused theoretical perspectives makes it difficult to understand and explain how and why interventions succeed or fail, “thus restraining opportunities to identify factors that predict the likelihood of implementation success and develop better strategies to achieve more successful implementation” [[26]: 1]. Theory-based research should be used to develop a more complete understanding of factors which influence healthcare professional’s behavior, with the goal to better inform the design of behavior change interventions [27–29]. Specifically, individuals’ decisions, primarily physicians’ decisions, are key to adopting a new practice; therefore, understanding the cognitive mechanisms underlying behaviors is critical to developing effective interventions [29].

Currently, the amount of empirical evidence on the use of theory in de-implementation is low, which limits knowledge about which specific factors influence de-implementation, the relevant barriers and facilitators, and which interventions are successful at reducing low-value care [22]. Therefore, there is a need for a systematic examination of the literature to identify and characterize the current body of research on the use of theory and frameworks in de-implementation. Through our review, we aimed to describe and synthesize the theories and frameworks, their use and application, and identify gaps in research. A recent review did much work to contribute to this literature, providing foundational analysis on the use of theories, models, and frameworks for the reduction of low-value care [15]. Our review builds on this work by identifying an additional 41 articles in which theory is used to understand the phenomenon of de-implementation; with the majority published in the last 2 years. As such, our scoping review adds breadth to the existing knowledge base, including a broad spectrum of theories and frameworks, providing comprehensive analysis and insight to this critical area of practice change.

## Methods

We selected a scoping review as the most appropriate approach as de-implementation research is relatively new, and little is known about the use of theory and frameworks in this area. We used Arksey and O’Malley’s [30] methodology for scoping reviews with consideration of recent perspectives on the methodology [31] and the Preferred Reporting Items for Systematic Reviews and Meta-Analysis Extension for Scoping Reviews (PRISMA-ScR) to guide the conduct and reporting of this review [32].

### Search strategy and data sources

With the assistance of a medical librarian, we conducted a comprehensive search of MEDLINE, EMBASE, CINAHL, and Scopus using the search terms listed in Table 1. We searched these databases from inception to June 2021. Based on the rationale for the study, the research team collaboratively determined the search strategy, selected the databases, and developed the search term criteria.

**Table 1:** Search terms

Concept	Search terms
**Value**	inappropriat* or overus* or unnecessary or ineffective or misus* or “do not do” or low-value or “low value” or obsole*
**Action**	reallocat* or relinquish* or re-apprais* or re-prioritiz* or redeploy* or revers* or decommission or declin* or delist* or abandon* or reassess* or replac* or disadopt* or defund* or de-adopt* or deadopt* or de-implement* or deimplement* or disinvest* or decreas* or discontinu* or withdraw* or stop* or reduc*
**Venue**	healthcare or “health care” or technolog* or device* or intervention* or health practi?e or medical or procedur* or drug* or medication*
	OR “choosing wisely”
**Theory**	theor* or framework or conceptual*

The search terms were selected based on a review of the existing literature and Niven et al.’s terminology findings [12]. Niven et al. found that “disinvest*” was the most common term used in their sample but advocated for the terms “de-adoption” and “de-implementation” to brand the process of reducing or eliminating low-value clinical practices as they felt these terms had a more general connotation and are natural antonyms of adoption and implementation [12]. In addition, the growth of Choosing Wisely campaigns (since its inception in 2012) and increased health services research aligned with these campaigns have necessitated the inclusion of related terms. The search strategy was developed iteratively and tested before the final search was conducted. The 43 search terms were selected to maximize the search capability to facilitate a fulsome search for this review.

### Eligibility criteria

All English language articles which explicitly used existing theory or frameworks to understand and/or explain influences on efforts to reduce low-value care were included. We only included empirical studies as we wanted to explore the application of theory to the practice of de-implementation. We excluded articles which focused only on identifying low-value care, testing the validity of Choosing Wisely, or measuring the use of low-value care. Systematic and scoping reviews were hand searched to identify any additional potentially relevant articles.

Studies were eligible for inclusion if they were categorized in Nilsen’s 2015 taxonomy of implementation science theories and frameworks as influencing outcomes [26]. This category consists of three types of theories or frameworks: (1) determinant frameworks, (2) classic theories, and (3) implementation theories [26]. We decided to focus on these categories as these are the types of theories and frameworks that are useful to the identification of potential barriers and enablers, and this area of knowledge and understanding is currently underdeveloped in de-implementation research.

Articles were excluded if they did not use theory to understand or explain the reduction of a low-value care. Studies were also excluded if they exclusively used quality improvement theory or methodology and did not also use theory from the category listed above. All study types were included in this scoping review.

### Data collection and extraction

The title and abstract screening process was conducted by one reviewer (GP) in consultation with a second reviewer (NS). A calibration exercise was conducted with a sample of three articles per reviewer prior to the title and abstract screening. Full-text screening was conducted by three reviewers (GP, NS, and TR) with a fourth reviewer (WB) checking a random 10% sample of articles to ensure reliability. Discrepancies at both levels of screening were discussed and resolved collaboratively. The data collection worksheet used for the full-text review was iteratively designed by the research team. The extraction worksheet was piloted with three included articles by three members of the research team (GP, NS, and TR). The worksheet was revised based on the results of the pilot review. Data was collected on the article characteristics, details of theory/framework selection and use, rationale and application, target low-value care, and level of behavior or practice change (e.g., individual or organization). These data were entered into an excel spreadsheet for synthesis and reporting.

### Data analysis

The data was analyzed by three research team members (GP, NS, and TR). We included two implementation science frameworks in the analysis: Nilsen’s categorization of implementation science theories and frameworks [26] and Birken’s categorization of use of theories and frameworks [33]. Numerical summary analysis and qualitative descriptive analysis was conducted, and discrepancies were discussed and resolved collaboratively. As the focus of this review was to identify and characterize theories and frameworks, their use and application in de-implementation research, and to identify gaps in research, we did not conduct a quality appraisal of the included studies.

## Results

**Fig. 1:**
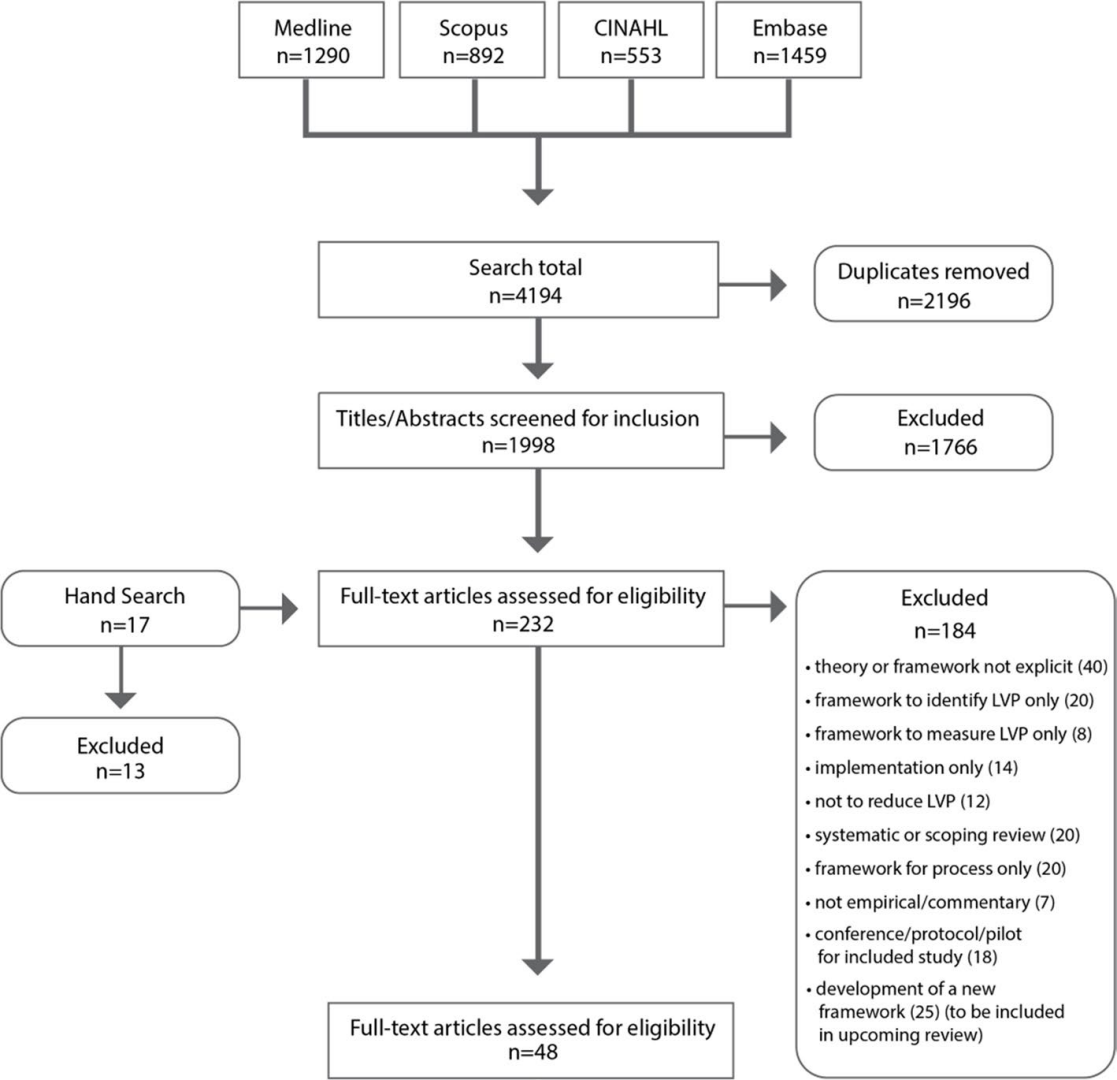
PRISMA diagram of the article selection process

The database searches identified 1998 articles (after duplicates were removed) for which the titles and abstracts were screened for inclusion. Of these, 232 articles were selected for full-text screening and 48 articles [22, 34–80] were included for this review. See Fig. 1 for the PRISMA diagram representing the complete article selection process.

### Study characteristics

Included studies were published between January 2010 and June 2021. The number of publications increased yearly: 7 articles were published in 2010‑2015 and 41 articles were published in 2016‑2021; with more than 60% of the articles published in the last 2.5 years (*n* = 30). The majority of articles were published by researchers in the USA (*n* = 14), the UK (*n* = 7), Canada (*n* = 7), Australia (*n* = 7), Ireland (*n* = 3), the Netherlands (*n* = 3), and Denmark (*n* = 2). The remainder of articles was published by researchers from Sweden, Malta, Iran, Switzerland, and China.

All articles reported on empirical studies with the majority using mixed methods (e.g., surveys, questionnaires, time series practice rate comparisons) (*n* = 25) or qualitative (*n* = 23) designs.

Of the 14 studies reporting on the use of an intervention to de-implement the low-value practice, ten studies found a reduction in the low-value care, two studies reported mixed results and two studies reported that the intervention was not effective in reducing the low-value care. Lin, Coffin, and O’Sullivan [42] used the TDF to identify barriers and enablers of target behaviors in a pilot study intended to reduce inappropriate low back pain (LBP) care. The results showed a dramatic reduction in guideline discordant LBP imaging referrals—reduced from 18/44 patients to 2/46 patients, and the authors noted that the improvements were “generally coherent with our analysis of barriers and enablers and the interventions we developed in response to these, providing support for this approach” [[42]:12].

### Theories and frameworks

Theories are “based on concepts or ideas that characterize a particular phenomenon and propositions or relationships that link the concepts” while frameworks “use concepts and relationships to provide a frame of reference, organize and focus thinking and assist interpretation” [[81]: 3-4]. Nilsen’s taxonomy includes three types of theories and frameworks used to understand and/or explain influences on outcomes: (1) *determinant frameworks* (frameworks that describe determinants believed to influence implementation outcomes), (2) *classic theories* (theories borrowed from other fields, e.g., psychology or sociology), and (3) *implementation theories* (theories developed by researchers for use in implementation science) [26]. Table 2 shows the breakdown of theories and frameworks identified by our review according to Nilsen’s taxonomy [26]. The majority of included articles used determinant frameworks (*n* = 27), followed by classic theories (*n* = 14), and implementation theories (*n* = 7). Thirteen of the included studies used secondary or multiple theories or frameworks, with the Behavior Change Wheel being the most common secondary framework (*n* = 5).

**Table 2:** Theories or frameworks

Category	Theory or framework		Number of studies (***n*** = 48)
Determinant frameworks	Theoretical Domains Framework (TDF) [82]	The TDF was developed to make behavior change theories more accessible to implementation researchers. The revised TDF consists of 84 constructs sorted into 14 domains which can be used to identify determinants of behavior and potential intervention.	22
	CFIR Consolidated Framework for Implementation Research (CFIR) [83]	The CFIR is an implementation science framework which can be used to identify determinants that may impact implementation. The CFIR can also be used to support design, evaluation, and implementation of intervention.	4
	Tailored Implementation for Chronic Disease (TICD) framework [84]	The TICD incorporates items from other commonly used frameworks, e.g., CFIR and TDF. The aim of this framework is to provide an accessible checklist to identify determinants that may impact implementation.	1
Classic theories	Theory of Planned Behavior (TPB) [85]/Theory of Reasoned Action (TRA) [86]	The TPB is a psychological theory that states that attitude, subject norms, and perceived behavioral control are antecedents to intention which is an antecedent to behavior. The TRA purports that intention to perform a behavior is the main predictor of that behavior.	7
	Fuzzy Trace Theory [87]	FTT is a cognitive theory which can be used to predict reasoning and decision-making.	2
	Lewin’s Change Theory [88]	Lewin’s Change Theory is a social psychology theory that purports that behavior is a dynamic balance of driving forces and resisting forces.	1
	Dual Processing Theory [89]	Dual process theory posits that two memory systems are involved in decision making. The first system is intuitive and relies on heuristics, the second system is analytical and deductive.	1
	Regulatory Fit Theory [90]	The regulatory fit theory proposes that individuals experience a state of regulatory fit when the approach to achieving a goal aligns with the goal orientation.	1
	Cognitive Dissonance Theory [91]	Cognitive dissonance theory posits that individuals have an inner drive to hold their cognitions and behaviors in harmony and avoid dissonance.	1
	Empowerment Theory [92]	Empowerment theory posits that work environments that provide employees with resources, support, and opportunities to learn promote empowerment.	1
Implementation theories	Normalization Process Theory (NPT) [93]	The NPT provides a framework for understanding and evaluating the processes by which interventions are embedded into everyday work and sustained.	4
	COM-B Model [94]^a^	The COM-B Model posits that capability, opportunity, and motivation interact to influence behavior.	3

^a^In this categorization scheme [26], the COM-B, a model that is predicated on multiple behavior change theories, is categorized as an implementation theory

The Theoretical Domains Framework (TDF)—a framework that harmonizes psychological and organizational theories—was the most commonly used determinant framework (*n* = 22), followed by CFIR (*n* = 4) and TICD (*n* = 1). The Theory of Planned Behavior (TPB) was the most frequently used classic theory (*n* = 6), followed by cognitive/decision-making theories, such as Fuzzy Trace Theory, Change Theory, Cognitive Dissonance Theory, and Dual Processing Theory (*n* = 5). The Theory of Reasoned Action, Empowerment Theory, and Regulatory Fit Theory were all used in one study each. Implementation theories were identified the least frequently, with the Normalization Process Theory (NPT) used by four studies and the COM-B Model used as the primary theoretical approach in three studies.

All of the 14 domains in the TDF were identified in the studies. Five domains were identified in the majority of these studies: environmental context and resources (*n* = 17), social influences (*n* = 17), knowledge (*n* = 16), beliefs about consequences (*n* = 13), and social/professional role and identity (*n* = 12). Emotion (*n* = 6), intention (*n* = 4), and optimism (*n* = 3) were identified least frequently. Three studies identified barriers and/or facilitators for all fourteen domains. Patey et al. [65] used the TDF to study anesthesiologists’ and surgeons’ perceptions about routine pre-operative testing in low-risk patients. The authors detailed how they used the domains to identify factors that influence physicians’ decisions to order pre-operative tests. The study included a content analysis of physician statements and mapped the data to the relevant domains, identifying *beliefs about capabilities* and *social influences* as key barriers impacting the use of pre-operative testing.

All of the studies which used the TPB as their theoretical framework studied the reduction of inappropriate antibiotic use. The reporting in these studies was less prescriptive. The impact of patients on provider behavior was prominent, as measured by the construct of perceived behavioral control. Byrne et al. [60] used the TPB to identify barriers and facilitators through a questionnaire to measure factors contributing to self-reported antibiotic use within the community. The authors reported that perceived behavioral control, social norms, and the interaction between attitudes and beliefs and knowledge were all significant predictors of antibiotic use behavior.

Similar to the application of the TPB, the majority of the studies which used cognitive theories studied the inappropriate use of antibiotics (*n* = 3). These studies were predominately physician-focused as the theories use decision-making as the nexus of change. Studies that used cognitive theories reported that using rational approaches to influence perceptions, preferences, and expectations were the most effective ways to change behavior. In their study, Gupta and colleagues [63] explored decision-making and cognitive biases through physicians’ experiences of trying to abandon an outmoded clinical practice. The authors extended Becker and Lewin’s theories by depicting physician learning as a multi-directional process of change which occurs on a continuous spectrum, rather than unidirectional and linear [63].

The studies which used implementation theories—Normalization Process Theory and COM-B Model—predominately focused on prescribing practices (*n* = 6). The NPT is primarily used to understand how an intervention can become embedded or routinized and was used in three of the included studies to develop an intervention or evaluate a process, as opposed to identifying barriers to behavior change. West and Cordina utilized the Normalization Process Theory (NPT), to develop their education intervention to reduce inappropriate antibiotic use [55]. The authors stated that the NPT is ideally suited to develop embedded interventions as it is a theory that is directed toward healthcare professionals and supports efforts to implement interventions into routine work practices. Their intervention was found to have significantly enhanced adherence to prescribed short-term antibiotics and reduced waste.

The COM-B Model was reported to be a useful theoretical perspective and elucidated factor for all three constructs—capability, opportunity, and motivation for three of the included studies.

### Use of theory or frameworks

**Fig. 2:**
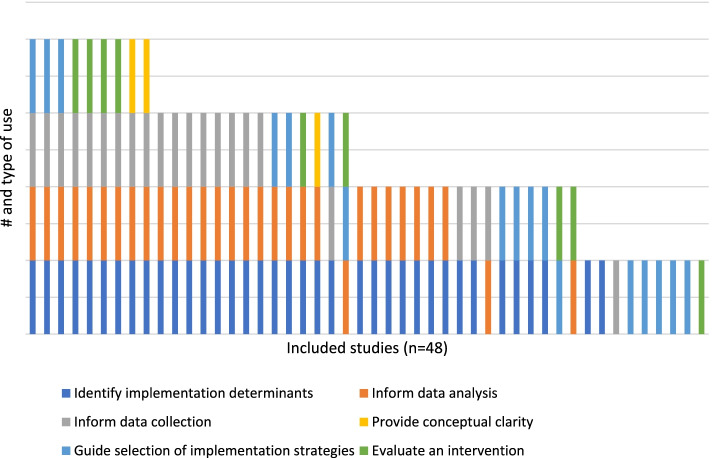
Use of theory or framework

In their international survey of implementation scientists, Birken et al. [33] categorized uses for theory in implementation science. Aligning with the top categories identified by Birken et al. [33], our review found that theories and frameworks were used most frequently to identify determinants (e.g., barriers and facilitators) (*n* = 37), inform data analysis, (*n* =3 1), inform data collection (*n* = 22), guide selection of implementation strategies (*n* = 17), evaluate an intervention (*n* = 9), and provide conceptual clarity (*n* = 3) (Fig. 2). Identifying determinants and data analysis were the most common combination of uses in the included articles (*n* = 29).

### Application of theory or frameworks

#### Individual-level application

All of the included articles used theory to address the reduction of low-value care at the individual level. Physician’s behavior was the focus of the majority of articles (*n* = 34), followed by behaviors on the part of patients (*n* = 12), nurses (*n* = 12), pharmacists (*n* = 6), other healthcare providers (*n* = 6), and managers/administrators (*n* = 2). Seven studies examined both healthcare provider and patient behavior.

#### Low-value care studied

**Fig. 3:**
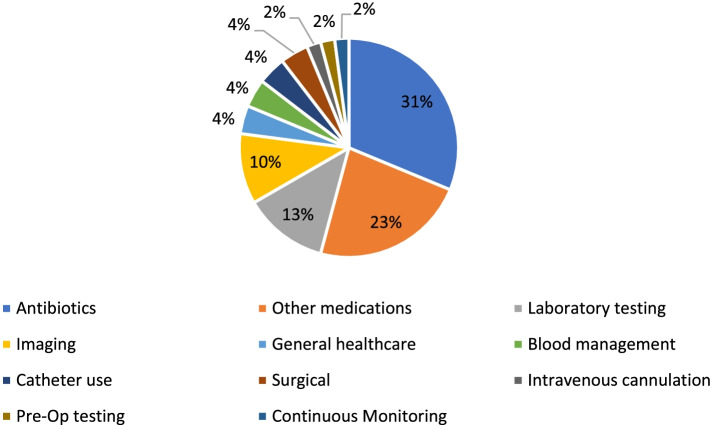
Low-value care studied

Reducing inappropriate antibiotic use was the most common low-value care studied (*n* = 15) followed by the reduction of other medications (*n* = 11) (Fig. 3). Reducing inappropriate imaging practices was addressed by six studies. Additional practices studied were laboratory tests, catheter use, intravenous cannulation, pre-operative testing, surgical procedures, continuous monitoring, and blood management practices. Two papers provided general applications of theory or frameworks which could be used to study a variety of low-value care.

Studies that focused on reducing inappropriate medications (antibiotics and others), used a variety of approaches to address the issue. The majority of the studies that addressed antibiotic misuse aimed to reduce inappropriate prescribing (*n* = 9); followed by studies which aimed to reduce the length of antibiotic use (*n* = 3), reduce patient misuse (*n* = 2), and improve hospital antimicrobial stewardship programs (*n* = 1). For studies which focused on reducing inappropriate prescribing for other medications, four studies addressed deprescribing; four studies addressed inappropriate prescribing, and three studies approached reduction through both prescribing and deprescribing.

## Discussion

These findings provide important insights into the emerging research area of de-implementation, and how theoretically based work is approaching this critical practice change. This review affords an up-to-date and comprehensive collection of theories and frameworks, and an analysis of how these theories and frameworks are used. This section offers a detailed discussion of the key findings, application, and gaps.

While the number of theory informed articles on this topic has steadily increased—over 60% of all included studies were published in the last 2.5 years—these numbers are only a small proportion of the studies published about reducing low-value care. This observation corroborates Niven et al.’s [12] finding that the majority of de-implementation efforts are likely atheoretical. Despite this low number, the included articles provide valuable insight into the maturing use of theory and frameworks in de-implementation and the dominant perspectives being used.

The TDF was the most commonly used framework in the included studies. Nilsen et al. noted that the use of TDF and TICD reinforces the importance of both individual and contextual determinants to de-implementation efforts [15] and results of the included studies align with this assertion. The majority of studies that used TDF identified individual—*knowledge* and *beliefs about consequences*—and contextual—*environmental context and resources*, and *social influences* determinants as key factors impacting de-implementation. The popularity of the TDF may be explained by the fact that it is useful for researchers in light of the myriad options available to them [10].

Our results also show a prevalence of psychological theories, particularly the Theory of Planned Behavior; this aligns with implementation science research where social cognitive theories have been the dominant theories used to understand clinical behavior change [26]. These classic theories, which typically only describe mechanisms and change but do not prescribe how to bring about change [15], are well established and have an abundance of empirical support in primary research. The prevalence of these theories also corresponds with the notion that it is the healthcare providers who ultimately prescribe or perform the low-value care and therefore the responsibility lies with the individual [21, 95]. In 2008, Godin et al. [29] published a systematic review that investigated the application of different social cognitive theories used to study healthcare professional’s behavior. Similar to our results, their review found that the TPB was the most commonly used theory, and the included studies reported between 25% and 34% of the variance in behavior was explained by the theory [29].

Nilsen et al. [96] detailed a novel perspective in their 2012 paper *Creatures of habit: accounting for the role of habit in implementation research on clinical behaviour change.* While this paper is not explicitly about de-implementation, the authors raise key questions about the utility of psychological theories, based on intention, to study behavior change, particularly in the context of stopping an existing practice. The authors acknowledged that social cognitive theories offer insight into analytical processes, but do not address repeated behaviors that do not require decision-making processes and noted that much of a physician’s daily work and interactions occur in unvaried settings and this behavior is guided more by habit than intention. These arguments also align with an observation made by Godin et al. [29] based on the findings of their systematic review of the use of social cognitive theories: that habit is a missing component to understanding healthcare professional’s behavior. Nilsen et al. [96] draw attention to dual processing models which detail two distinct modes—automatic and intentional—for reasoning and information processing. The lack of incorporation of automatic processing—fast, intuitive, and often heuristic-based—into behavior change interventions could be key to increasing uptake of evidence-based practice [96]. The authors stated that interventions that impact habits—discontinuation of harmful or ineffective habits or the development of beneficial habits—could be key to effecting behavior change [96]. This critique of the use of psychological theories in behavior change research may be reflected in our findings as increasingly determinant frameworks are being used in de-implementation research.

Our results align with Birken et al.’s [33] findings regarding the use of theory in implementation science. The implementation scientists surveyed in their study reported that they used theory primarily to identify facilitators and barriers which aligns with our findings. Seventy-seven percent of the implementation scientists in Birken et al.’s study reported that they used theory for data collection which is a significantly higher rate than shown in our study (46%). Sixty-five percent of our included studies used theory or frameworks to inform data analysis which also aligns with the 60% of participants in Birken et al.’s survey.

Similar to Nilsen et al.’s scoping review of the use of theory in de-implementation research [15], our results show that few studies examine the impact of patients on efforts to de-implement low-value care. Twelve of the 48 included studies considered patient impacts, with only five of these studies focusing on patient behavior alone. While the number of studies that include patients is low, analyzing these studies on the timeline shows that 11 of these 12 studies which examined patient factors were published in the last 2 ½ years (Nilsen et al.’s [15] inclusion period concluded mid-2018). Prusaczyk et al. argue that the impact of patients (community members and consumers, together “stakeholders”) is a unique and significant factor for de-implementation [17]. Therefore, while the number of studies is low, the trend implies that de-implementation research is increasingly including patient factors to understand and increase the effectiveness of efforts to reduce low-value care.

Many of the included studies not only explicitly report that a lack of knowledge or education is a key barrier to reducing low-value care but also acknowledge that knowledge is necessary, but not sufficient to effect change. This finding aligns with recent work on the effect of passive versus active change interventions which suggest that passive interventions, such as education sessions or the publication of guidelines or “do not do” lists alone, do not appear to be effective to change healthcare provider behavior to reduce low-value care [10, 27, 97–101]. A 2015 study of early trends for seven Choosing Wisely recommendations concluded that the recommendations alone were not enough to produce significant changes in practice and that active interventions, such as financial incentives, data feedback, and systems-level interventions increase the effectiveness of practice change [100].

Three studies cited the Choosing Wisely campaigns as the motivation for the study, with an additional 3 studies referencing the campaigns. Other studies have identified a significant amount of research published in recent years addressing the Choosing Wisely campaigns and efforts to reduce practices identified on their “do not do” lists. This suggests to us that research specific to addressing Choosing Wisely recommendations is largely atheoretical at this point. Choosing Wisely campaigns are premised on the idea that the responsibility to reduce unnecessary practices is to be shared between physicians and patients with physicians as the stewards of healthcare resources, charged with ensuring that care is appropriate and sustainable [102, 103]. This individual-level focus aligns with all of the studies included in this review and the type of theories used could benefit Choosing Wisely inspired efforts to reduce low-value care. Grimshaw et al. developed a Choosing Wisely De-implementation Framework (CWDIF) in an effort to address this gap [10]. Models and frameworks developed specifically to support de-implementation is the focus of our subsequent scoping review, which will complement and expand the findings of this review.

## Limitations

This study has several limitations. As Niven et al. [12] and other researchers have noted there are many different terms used to refer to the process of reducing or eliminating low-value care; this study may have omitted some terms from the search criteria and may therefore have excluded relevant studies. In addition, articles published in languages other than English were not included due to a lack of resources. As is typical with scoping reviews, we did not assess the quality of the included articles. In addition, this review used implementation science as a basis for data collection and analysis. While this approach is debated in the literature [2, 10, 15, 16], we argue that this approach offers a solid foundation from which to study emerging research in de-implementation. This review focused on the use of existing theories and frameworks in de-implementation research. There is a growing body of literature that has produced frameworks and models to understand or explain efforts to reduce low-value care. We acknowledge the importance of this emerging literature, and this area of research is the focus of our subsequent scoping review.

## Conclusion

This review adds value to de-implementation research through our use of enhanced rigor and a broad and comprehensive search. We systematically examined and characterized the theories and frameworks, their use and application, and gaps in the literature. These insights into how and why theories and frameworks are being used to understand and explain factors that influence efforts to reduce low-value care can inform research priorities, a systematic review of the literature and the development of targeted strategies and interventions. As healthcare organizations, hospitals, and healthcare providers continue to address the reduction of low-value care, more interventions and studies will use theory and more data on this topic will be available to facilitate more comprehensive and in-depth studies.

## Data Availability

The datasets supporting the conclusions of this article are included within the article and its additional files.

## Abbreviation

CINAHL: Cumulative Index to Nursing and Allied Health Literature
